# Xylazine Activates Adenosine Monophosphate-Activated Protein Kinase Pathway in the Central Nervous System of Rats

**DOI:** 10.1371/journal.pone.0153169

**Published:** 2016-04-06

**Authors:** Xing-Xing Shi, Bai-Shuang Yin, Peng Yang, Hao Chen, Xin Li, Li-Xue Su, Hong-Gang Fan, Hong-Bin Wang

**Affiliations:** 1 Department of Veterinary Surgery, College of Veterinary Medicine, Northeast Agricultural University, Harbin, Heilongjiang Province, People’s Republic of China; 2 Department of Veterinary Medicine, Jilin Agriculture Science and Technology College, Jilin, Jilin Province, People’s Republic of China; Rutgers University, UNITED STATES

## Abstract

Xylazine is a potent analgesic extensively used in veterinary and animal experimentation. Evidence exists that the analgesic effect can be inhibited using adenosine 5’-monophosphate activated protein kinase (AMPK) inhibitors. Considering this idea, the aim of this study was to investigate whether the AMPK signaling pathway is involved in the central analgesic mechanism of xylazine in the rat. Xylazine was administrated via the intraperitoneal route. Sprague-Dawley rats were sacrificed and the cerebral cortex, cerebellum, hippocampus, thalamus and brainstem were collected for determination of liver kinase B1 (LKB1) and AMPKα mRNA expression using quantitative real-time polymerase chain reaction (qPCR), and phosphorylated LKB1 and AMPKα levels using western blot. The results of our study showed that compared with the control group, xylazine induced significant increases in AMPK activity in the cerebral cortex, hippocampus, thalamus and cerebellum after rats received xylazine (*P* < 0.01). Increased AMPK activities were accompanied with increased phosphorylation levels of LKB1 in corresponding regions of rats. The protein levels of phosphorylated LKB1 and AMPKα in these regions returned or tended to return to control group levels. However, in the brainstem, phosphorylated LKB1 and AMPKα protein levels were decreased by xylazine compared with the control (*P* < 0.05). In conclusion, our data indicates that xylazine alters the activities of LKB1 and AMPK in the central nervous system of rats, which suggests that xylazine affects the regulatory signaling pathway of the analgesic mechanism in the rat brain.

## Introduction

Xylazine is exclusively used as a sedative, analgesic, and muscle relaxant in veterinary medicine, and is marketed as Rompun, Anased, Sedazine, Megaxilor, Paxman and Chanazine [1, 2]. Xylazine (N-(2,6-dimethylphenyl)-5,6-dihydro-4H-1,3-thiazin-2-amine) is an effective sedative and analgesic and has potential to use during surgical operations for pain relief to reduce discomfort and stress [1]. In animal experiments, xylazine is a component of the most common injectable anesthetic, ketamine-xylazine, which is used in rats, mice, hamsters, and guinea pigs [3]. Intravenous administration results in deep dose-dependent sedation, characterized by somnolence and low head carriage in horses [4]. Xylazine is a strong α2-adrenergic agonist whose effects are mediated via stimulation of central α2-receptors. α2-adrenergic stimulation decreases the release of norepinephrine and dopamine in the central nervous system (CNS) resulting in sedation, muscle relaxation, and decreased perception of painful stimuli. Moreover, its actions may also be involved in cholinergic, serotonergic, dopaminergic, α1-adrenergic, histaminergic, or opiate mechanisms [5]. Xylazine is absorbed, metabolized, and eliminated rapidly. It diffuses extensively and penetrates the blood brain barrier, as expected due to the uncharged, lipophilic nature of the compound [6]. When xylazine and other α2-adrenergic receptor agonists are administered, they distribute throughout the body within 30 to 40 minutes. The sedative and analgesic effects of xylazine inhibit the transmission of neural impulses in the CNS [7]. As an agonist, xylazine leads to a decrease in neurotransmission of norepinephrine and dopamine [6].

In recent years, there has been increasing interest in the study of the molecular and cellular mechanisms underlying general anaesthesia. Among the different mechanisms, research on the regulation of pain signaling by adenosine 5’-monophosphate activated protein kinase (AMPK) has become a main research focus [8]. The heterotrimeric protein AMPK plays a critical regulatory role in cellular energy homeostasis and organismal metabolism [9]. This serine/threonine kinase is formed by an α catalytic subunit and two regulatory subunits, β and γ [10, 11]. In mammals, the catalytic α subunit of AMPK has two isoforms, α1 and α2 [12]. AMPKα1 and AMPKα2 can be activated in response to pharmacological agents in a Liver kinase B1 (LKB1)-dependent manner [13]. LKB1 is a tumor suppressor gene mutated in the inherited cancer disorder Peutz-Jeghers syndrome [14]. In addition to nucleotide binding, phosphorylation of Thr172 at AMPK is required for its activation, and several groups have demonstrated that LKB1 is constitutively active and phosphorylates AMPK at Thr172 of the α subunit [15, 16]. Genetic studies of tissue-specific deletion of LKB1 have revealed that LKB1 mediates the majority of AMPK activation in nearly every tissue type examined to date [17, 18]. AMPK can also be phosphorylated in response to calcium flux via calcium/calmodulin-dependent kinase 2 (CAMKK2) kinase [19, 20].

When activated by LKB1, AMPK elicits its effects by regulating the activities of key metabolic enzymes via suppression of the mammalian target of rapamycin complex 1 pathway [21, 22]. Recent evidence suggests that peripheral pain plasticity is promoted and potentially maintained via changes in translation control that are mediated by mammalian target of rapamycin complex 1 and mitogen-activated protein kinase. Due to its significant sensor role in modulating pathways in catabolic processes, AMPK activation is correlated with peripheral nerve injury- and incision-induced pain and the blockade of the development of chronic pain following surgery [8]. AMPK gene expression levels have been studied in electroacupuncture-induced analgesia [23]. Moreover, AMPK plays a unique role for drug development in the kinase area for pain because it is pharmacologically manipulated via activation rather than inhibition [8]. Altogether, the physiology, pharmacology, and therapeutic opportunities surrounding AMPK make it an attractive target for novel intervention for pain.

To the best of our knowledge, there are no data available on the gene expression patterns or protein levels of AMPK and its upstream regulator LKB1 in different brain regions of xylazine-treated rats. We measured the mRNA and protein levels of LKB1 and AMPKα in the cerebral cortex, cerebellum, hippocampus, thalamus and brainstem of rats following intraperitoneal injection of xylazine. Hence, to elucidate the mechanisms involved in intraperitoneal xylazine administration, the goal of the present study was to determine whether the LKB1-AMPK pathway is involved in xylazine-induced sedation in the CNS which would lead us to further understanding in the field of veterinary anaesthesia and analgesia.

## Materials and Methods

### Ethics statement

All animal work was conducted according to the guidelines for the care and use of experimental animals established by the Ministry of Science and Technology of the People's Republic of China (Approval number: 2006–398), and was approved by the Laboratory Animal Management Committee of Northeast Agricultural University. All efforts were made to minimize animal stress/distress.

### Animals and experimental protocol

Healthy male Sprague-Dawley rats (n = 30) weighing approximately 160–180 g were obtained from the Animal Center of Harbin Medical University (Harbin, China). Animals were housed at constant room temperature and maintained under a 12/12-h light-dark cycle. Rats were allowed free access to commercial pellets (Animal Center of Harbin Medical University, Harbin, China) for at least one week. All experiments were performed in rats that had been deprived of food for 24 h, but free access to water. After acclimatization, the rats were randomly assigned to control or xylazine groups. Six rats received intraperitoneal injection of saline (0.5 mL, control group) and were sacrificed 10 min later. Twenty-four rats in the xylazine group were further subdivided into four groups. After receiving an injection of xylazine (5.2 mg/kg diluted in 0.5 mL saline), the rats were sacrificed 10 min (Xyl1 group), 20 min (Xyl2 group), 40 min (Xyl3 group) or 60 min (Xyl4 group) respectively. Six rats from each group were euthanized by cervical dislocation. A midline incision on the scalp was made to fully expose the sutures on the dorsal surface of the skull. The occipital, parietal and temporal skull plates were quickly removed with the use of bone rongeurs. Then the brains were immediately removed and placed in ice-cold slurry of 0.9% (w/v) NaCl. Five brain structures were dissected under a microscope: cerebral cortex, cerebellum, hippocampus, thalamus and brainstem. Dissected tissues were immediately frozen in liquid nitrogen and stored at −80°C for pending analysis.

### Quantitative real-time polymerase chain reaction (qPCR)

Total RNA was isolated from the brain tissues using TransZol reagent (TransGen Biotech, Beijing, China) following the manufacturer’s instructions, and the quality was assessed by spectrophotometric absorbance at 260/280 nm. First-strand complementary DNA (cDNA) synthesis was performed with 0.5 μg of total RNA using the ReverTra Ace qPCR RT Master Mix with a gDNA Remover kit (Toyobo, Osaka, Japan) according to the instructions of the manufacturer. Quantitative real-time PCR was performed using the Thunderbird SYBR qPCR Mix (Toyobo) in a LightCycler 2.0 (Roche Applied Science, Penzberg, Germany) according to the manufacturer’s instructions. The procedure included 1 cycle of 95°C for 30 s, followed by 45 cycles of 95°C for 5 s, primer-specific annealing temperature for 20 s, and extension at 72°C for 20 s. At the completion of the run, melting curves were generated. Fluorescence was monitored at 530 nm. Specificity of primer combinations was confirmed by melting curve of the PCR products. The experiment was performed in three replicates for qPCR. The relative expression levels of mRNA were analyzed using the 2^−ΔΔCt^ method [24]. The primers (Table 1) used for amplification by qPCR were synthesized by Sangon Biotech (Shanghai, China). All other reagents used in this study were of analytic grade.

**Table 1:** Sequences of primers used for the quantitative real-time PCR.

Gene	GenBank number	Primer sequences (5′-3′)	Product size (bp)
LKB1	NM_001108069	Forward: AGCCAAGAGGTTCTCCATCC	114
		Reverse: CAGCGGTCCTTAGTGTCTGG	
AMPKα1	NM_019142	Forward: GAAGTCAAAGCCGACCCAAT	116
		Reverse: AGGGTTCTTCCTTCGCACAC	
AMPKα2	NM_023991	Forward: ATGATGAGGTGGTGGAGCAG	117
		Reverse: GTGAATGGTTCTCGGCTGTG	
β-actin	NM_031144	Forward: AGGGAAATCGTGCGTGACAT	163
		Reverse: CCTCGGGGCATCGGAA	

LKB1, liver kinase B1; AMPKα1, adenosine 5’-monophosphate-activated protein kinase α1; AMPKα2, adenosine 5’-monophosphate-activated protein kinase α2.

### Protein isolation and western blot analysis

Frozen tissues were homogenized in ice-cold radioimmunoprecipitation assay lysis buffer (Beyotime Biotechnology, Nanjing, China) containing protease and phosphatase inhibitors (Sangon Biotech) for 5 min, and then incubated on ice for 30 min. The homogenate was then centrifuged at 14,000 × g for 10 min at 4°C. After the supernatant was collected, protein concentrations of the supernatants were determined with the bicinchoninic acid protein assay kit (Beyotime Biotechnology) according to the manufacturer’s protocol. The supernatants used as protein samples were boiled at 100°C for 5 min with 5 × SDS sample buffer (Beyotime Biotechnology), which were equivalent to 50 μg of protein. Samples were subjected to sodium dodecyl sulfate polyacrylamide gel electrophoresis by using 10% (w/v) gel, followed by transfer onto nitrocellulose membranes using the Bio-Rad Wet Trans-Blot apparatus (Bio-Rad, Hercules, USA). Non-specific binding sites were blocked by incubation with 5% (w/v) non-fat dry milk freshly prepared in Tris-buffered saline containing 0.05% (v/v) Tween-20 (TBST) for 2 h at room temperature. The nitrocellulose membranes were then incubated with primary antibodies overnight at 4°C. After three washes with TBST, the membranes were incubated with appropriate secondary antibodies conjugated to horseradish peroxidase for 2 h at room temperature. After washing four times in TBST, the signals were visualized using enhanced chemiluminescence (ECL) detection reagents (Advansta, Menlo Park, USA). The bands were scanned using a Tanon 5200 Imaging System (Tanon Science & Technology Co., Shanghai, China) with a 16-bit camera, and quantified by densitometry. The primary antibodies were used at the following dilutions, and obtained from the indicated sources: anti-p-AMPKα (Thr172) diluted 1:3000 (Cell Signaling Technology, Beverly, USA), anti-AMPKα diluted 1:3000 (Cell Signaling Technology), anti-p-LKB1 (Ser428) diluted 1:3000 (Cell Signaling Technology), anti-LKB1 diluted 1:3000 (Cell Signaling Technology), and anti-β-actin diluted 1:5000 (Zhongshan Goldenbridge Biotech, Beijing, China). Secondary horseradish peroxidase-conjugated antibodies (Zhongshan Goldenbridge Biotech) were used at a dilution of 1:5000.

### Statistical analysis

All data are presented as the mean ± standard error of the mean (SEM). The mean mRNA expression ratio in the control group was designated as one. Statistically significant differences among the means were determined using one-way analysis of variance (ANOVA) followed by Tukey's post hoc tests. All calculations were performed using PASW Statistics 17 (SPSS Inc., Chicago, USA). A p value of < 0.05 was considered statistically significant.

## Results

### Xylazine alters mRNA levels of LKB1 in different brain regions of rats

**Fig 1:**
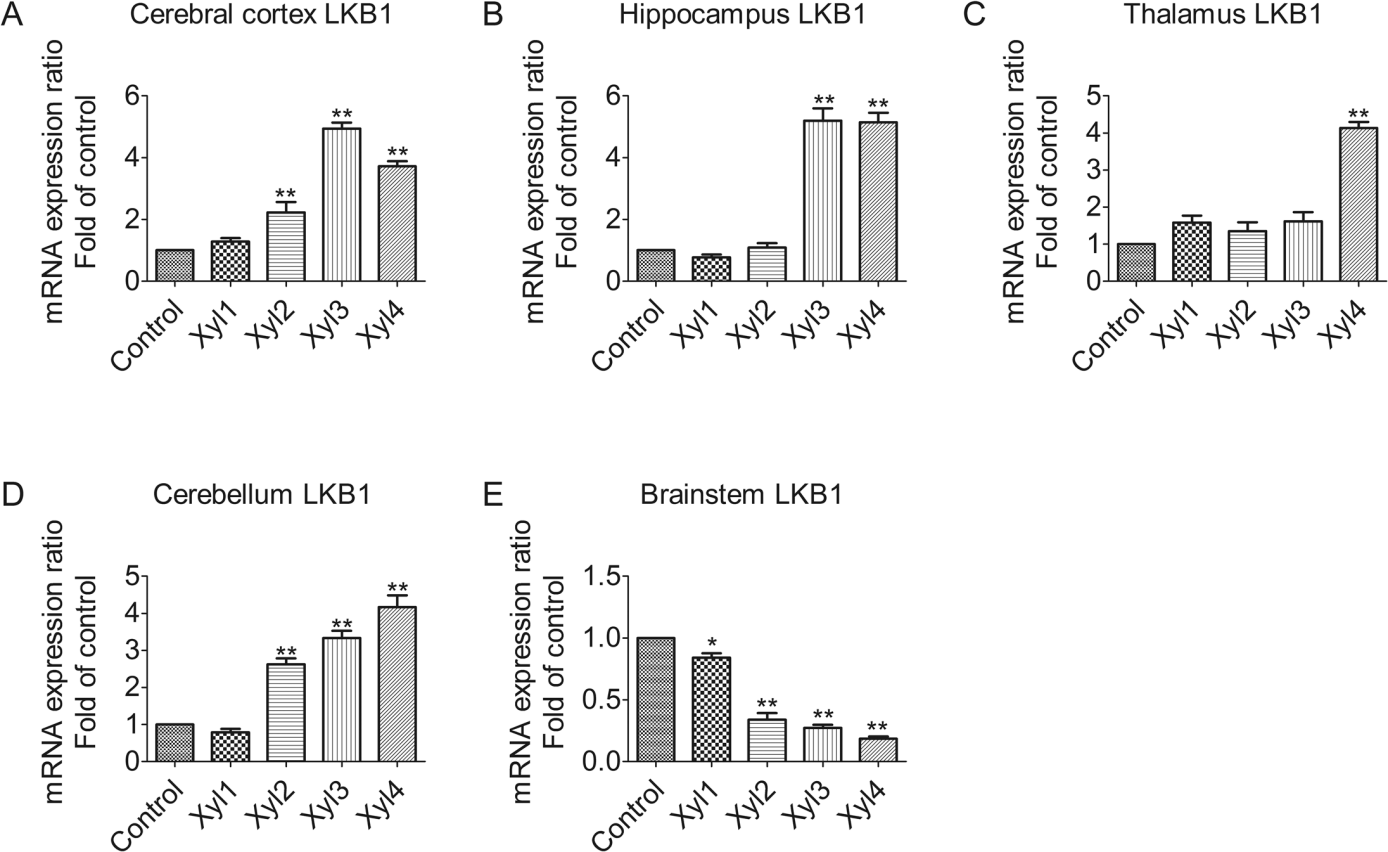
Effect of xylazine administration on the mRNA levels of LKB1 in rats. (A) Cerebral cortex, (B) Hippocampus, (C) Thalamus, (D) Cerebellum and (E) Brainstem. Rats received saline (0.5 mL) or xylazine (5.2 mg/kg) intraperitoneally and then were sacrificed 10, 10, 20, 40 or 60 min later for control, Xyl1, Xyl2, Xyl3 or Xyl4, respectively. Total RNA was isolated and subjected to real-time PCR analysis. Each value of the expression levels of LKB1 was normalized to the expression levels of β-actin, and the control value was set to one. Data are presented as the means ± SEM, n = 6. * P < 0.05, ** P < 0.01 vs control.

Quantitative real-time PCR analysis revealed that treatment with 5.2 mg/kg of xylazine altered the expression of LKB1 mRNA in different rat brain regions (Fig 1). However, the magnitude of the alteration varied with time. In the cerebral cortex, the level of LKB1 increased slightly at 10 min (Xyl1, 1.3-fold increase, P > 0.05) after drug administration compared with the control group, peaked at 40 min (Xyl3, 4.9-fold increase, P < 0.01), and then decreased rapidly, but remained elevated at 60 min (3.7-fold increase, P < 0.01, Fig 1A). In the hippocampus, the level of LKB1 began to increase markedly at 40 min (Xyl3, 5.2-fold increase, P < 0.01, Fig 1B). Similarly, thalamic levels of LKB1 mRNA increased at 60 min (Xyl4, 4.1-fold increase, P < 0.01, Fig 1C). The level of LKB1 decreased slightly at 10 min (Xyl1, 0.8-fold increase, P > 0.05) and then increased markedly (Xyl2, 2.6-fold increase, P < 0.01) at 20 min in the cerebellum, and this elevation became more marked as time increased (Xyl4, 4.2-fold increase, P < 0.01, Fig 1D). Unlike the previous four regions, there was a continuous downward tendency of LKB1 mRNA levels in the brainstem. A marked decrease in mRNA expression was observed at 60 min (Xyl4, 0.2-fold decrease, P < 0.01, Fig 1E).

### Xylazine alters the expression of AMPKα1 mRNA levels in different brain regions of rats

**Fig 2:**
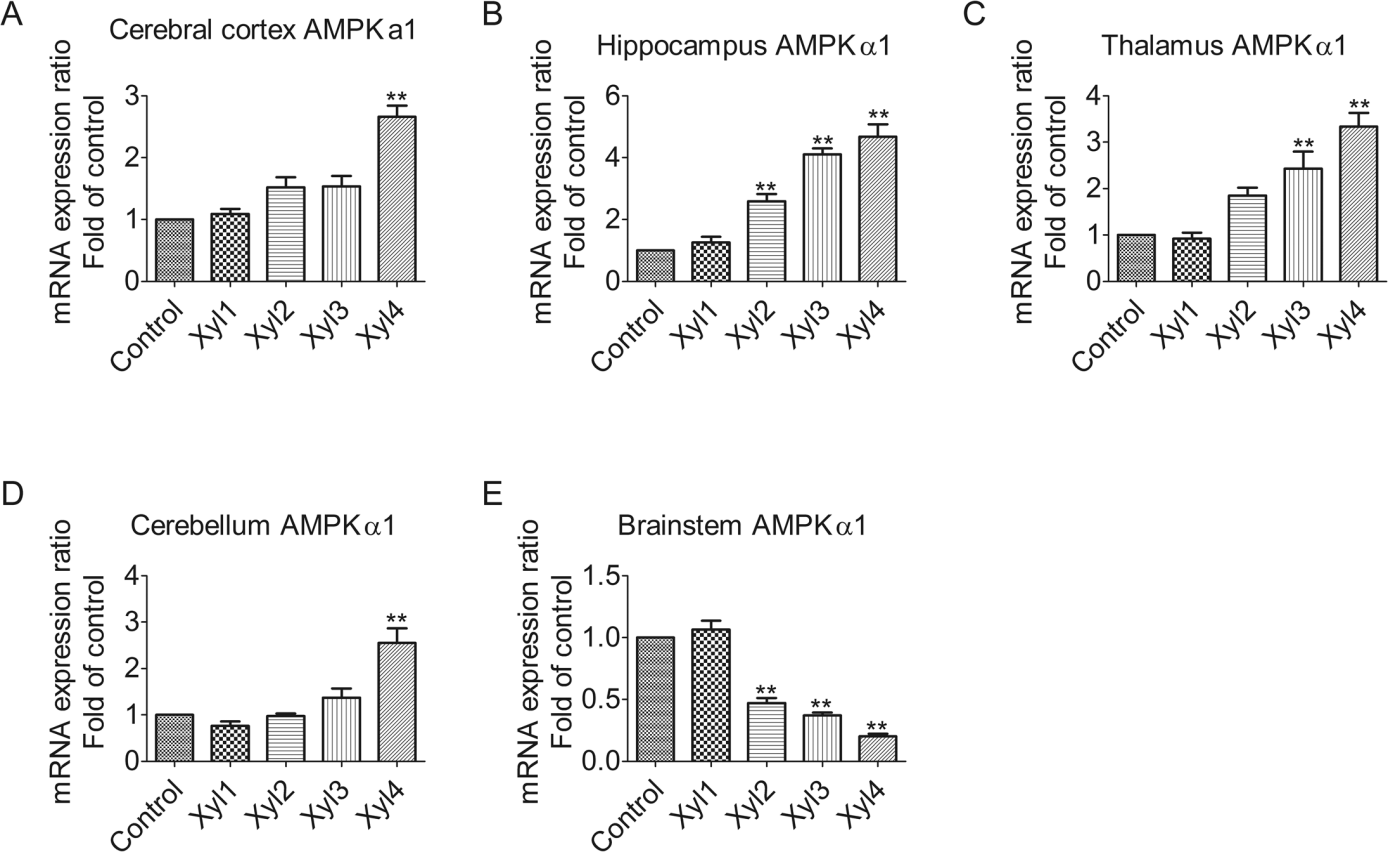
Effect of xylazine administration on the mRNA levels of AMPKα1 in rats. (A) Cerebral cortex, (B) Hippocampus, (C) Thalamus, (D) Cerebellum and (E) Brainstem. Rats received saline (0.5 mL) or xylazine (5.2 mg/kg) intraperitoneally and then were sacrificed 10, 10, 20, 40 or 60 min later for control, Xyl1, Xyl2, Xyl3 or Xyl4, respectively. Total RNA was isolated and subjected to real-time PCR analysis. Each value of the expression levels of AMPKα1 was normalized to the expression levels of β-actin, and the control value was set to one. Data are presented as the means ± SEM, n = 6. * P < 0.05, ** P < 0.01 vs control.

Changes in AMPKα1 mRNA levels in the rat brain were determined by qPCR (Fig 2). The mRNA levels for AMPKα1 were raised after rats were intraperitoneally injected with xylazine in the cerebral cortex. A significant elevation was observed at 60 min (Xyl4, 2.7-fold increase, P < 0.01, Fig 2A). The most obvious increase in mRNA expression was observed at 60 min (Xyl4, 4.7-fold increase, P < 0.01) in the hippocampus (Fig 2B). Whereas, in the thalamus, the level of AMPKα1 mRNA began to increase significantly at 40 min (Xyl3, 2.4-fold increase, P < 0.01). A further increase was seen at 60 min (Xyl4 3.3-fold increase, P < 0.01, Fig 2C). As shown in Fig 2D, however, no significant differences were observed among the control, Xyl1, Xyl2 and Xyl3 groups for the cerebellum, but Xyl4 showed increased (2.6-fold increase, P < 0.01) levels at 60 min. In contrast, xylazine treatment caused a marked downregulation of AMPKα1 mRNA expression at 20 min (Xyl2, 0.5-fold decrease, P < 0.01, Fig 2E) in the brainstem.

### Xylazine alters the expression of AMPKα2 mRNA levels in different brain regions of rats

**Fig 3:**
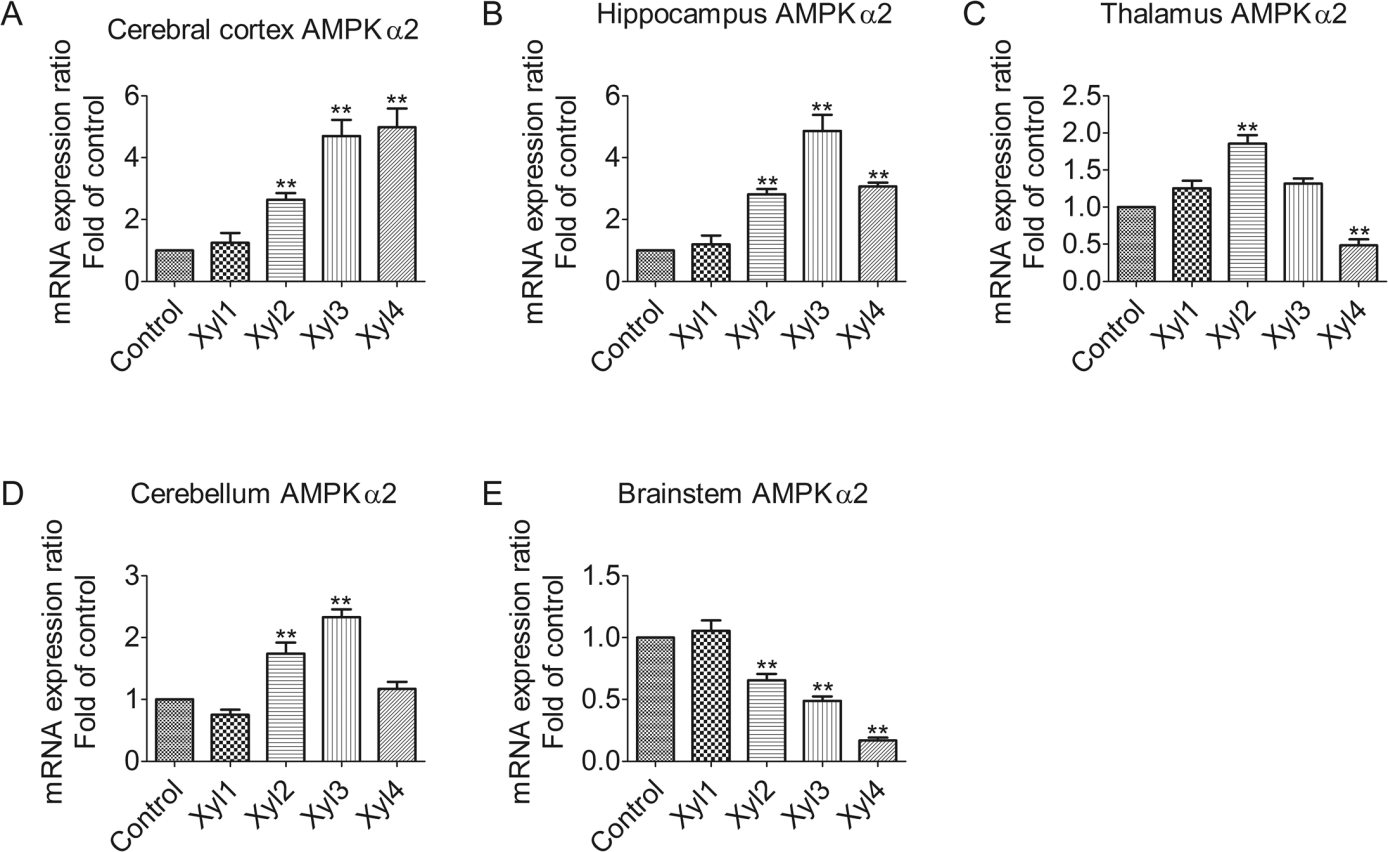
Effect of xylazine administration on the mRNA levels of AMPKα2 in rats. (A) Cerebral cortex, (B) Hippocampus, (C) Thalamus, (D) Cerebellum and (E) Brainstem. Rats received saline (0.5 mL) or xylazine (5.2 mg/kg) intraperitoneally and then were sacrificed 10, 10, 20, 40 or 60 min later for control, Xyl1, Xyl2, Xyl3 or Xyl4, respectively. Total RNA was isolated and subjected to real-time PCR analysis. Each value of the expression levels of AMPKα2 was normalized to the expression levels of β-actin, and the control value was set to one. Data are presented as the means ± SEM, n = 6. * P < 0.05, ** P < 0.01 vs control.

We examined the expression pattern of AMPKα2 among different brain regions (Fig 3). The qPCR analysis showed that the relative expression levels of AMPKα2 tended to increase with time in the cerebral cortex. The level of AMPKα2 mRNA was markedly elevated at 60 min (Xyl4, 4.9-fold increase, P < 0.01, Fig 3A). In the hippocampus, AMPKα2 mRNA was significantly upregulated at 40 min (Xyl3, 4.8-fold increase, P < 0.01), but declined at 60 min (Xyl4, 3.1-fold increase, P < 0.01, Fig 3B). Xylazine increased mRNA levels at 20 min (Xyl2, 1.8-fold increase, P < 0.01), with a subsequent decline at 60 min (Xyl4, 0.5-fold decrease, P < 0.01, Fig 3C) in the thalamus. In the cerebellum, the levels of AMPKα2 increased markedly at 20 min (Xyl2, 1.7-fold increase, P < 0.01) and reverted to control levels (Xyl4, 1.2-fold increase, P > 0.05, Fig 3D). Consistent with the results of AMPKα1 in the brainstem, the progressive attenuation of AMPKα2 mRNA was observed at 20 min (Xyl2, 0.7-fold decrease, P < 0.01, Fig 3E).

### Effect of xylazine on phosphorylated LKB1 levels in different brain regions of rats

**Fig 4:**
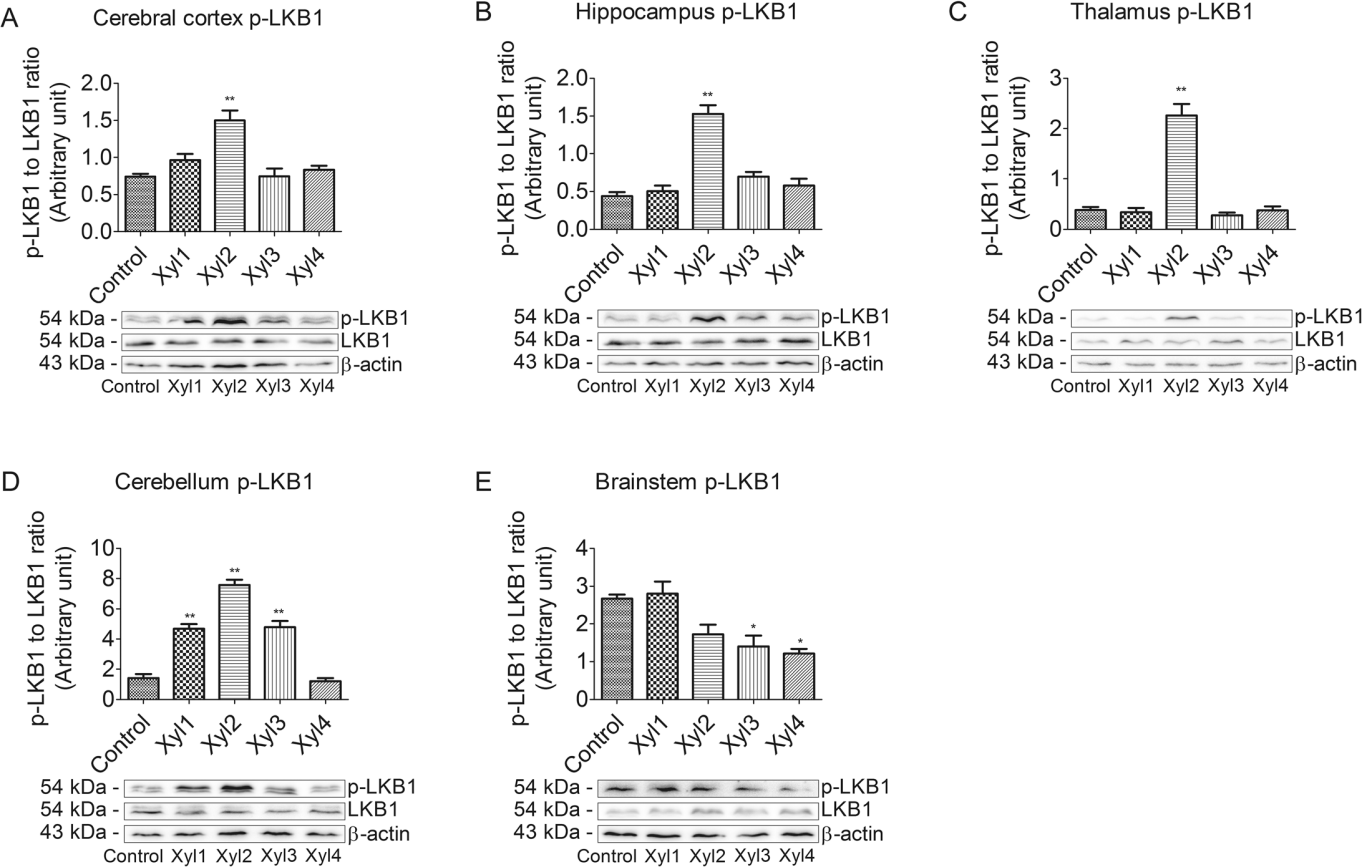
Effect of xylazine administration on the levels of phosphorylated LKB1 in rats. (A) Cerebral cortex, (B) Hippocampus, (C) Thalamus, (D) Cerebellum and (E) Brainstem. Rats received saline (0.5 mL) or xylazine (5.2 mg/kg) intraperitoneally and then were sacrificed 10, 10, 20, 40 or 60 min later for control, Xyl1, Xyl2, Xyl3 or Xyl4, respectively. Western blot analyses were performed with anti-LKB1 and anti-phospho-LKB1 (Ser428). Data for densitometry represent the mean ± SEM obtained from six independent series of Western blotting for each animal group and time point after the procedure. * P < 0.05, ** P < 0.01 vs control. Representative blots are shown below graph.

To investigate whether the LKB1/AMPK signaling pathway was involved in analgesia, we first examined the phosphorylation of LKB1 at Ser428 using western blot (Fig 4). In the cerebral cortex, compared with the control group, the phosphorylation of LKB1 was significantly increased in the Xyl2 group at 20 min after drug administration and recovered at 40 min (P < 0.01, Fig 4A). The change in LKB1 expression in the hippocampus or thalamus was similar to that in the cerebral cortex. However, the phosphorylated LKB1 levels increased at 20 min and decreased again at 30 min, while no significant changes were seen among the other groups in the hippocampus or thalamus (Fig 4B and 4C). The phosphorylation of LKB1 began to increase markedly at 10 min after xylazine administration (P < 0.01) and recovered at 60 min in the cerebellum of rats (Fig 4D). In the brainstem, phosphorylated LKB1 levels increased slightly at 10 min compared with the control group, and then began to decrease at 40 min (P < 0.05, Fig 4E).

### Effect of xylazine on phosphorylated AMPKα levels in different brain regions of rats

**Fig 5:**
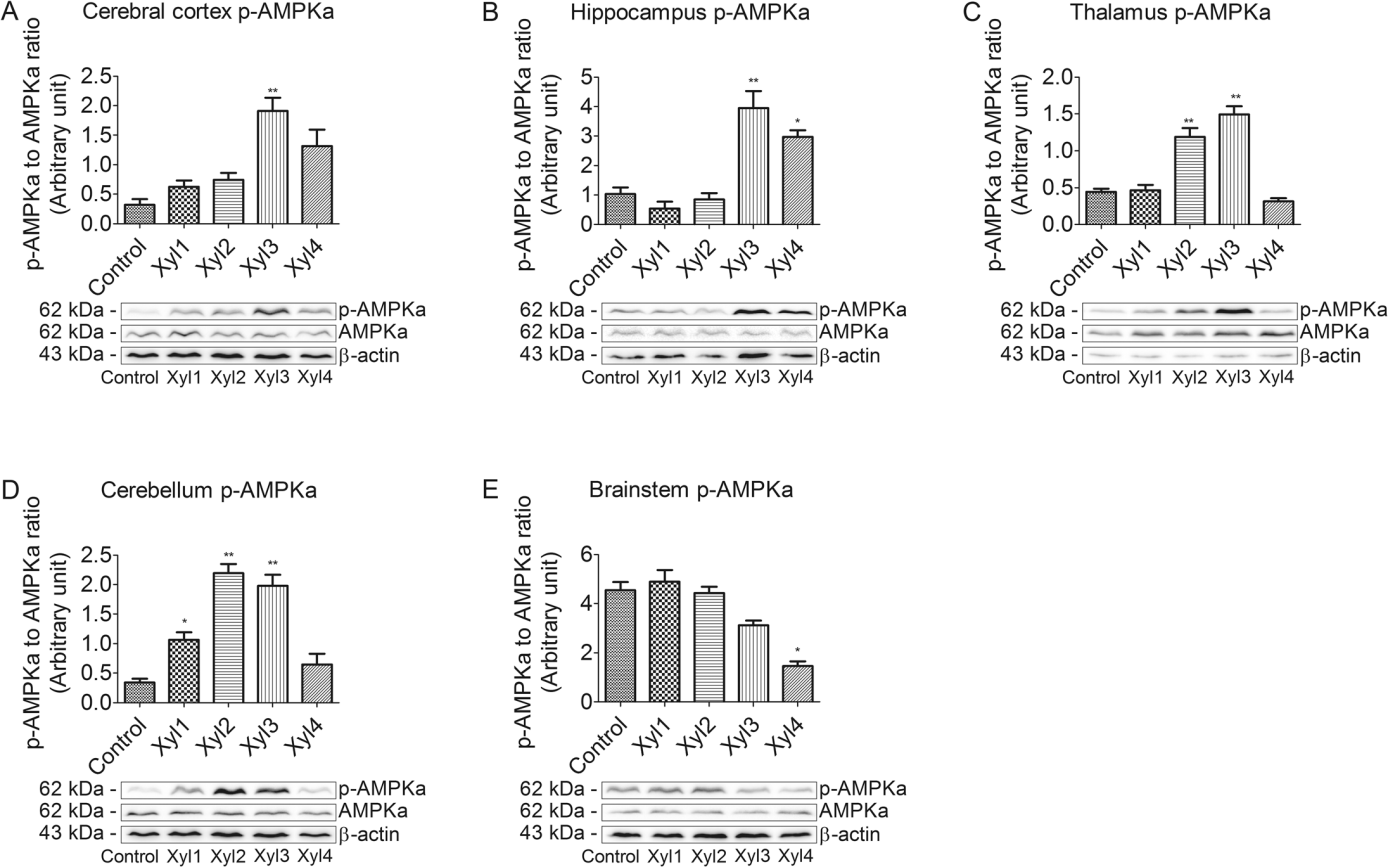
Effect of xylazine administration on the levels of phosphorylated AMPKα in rats. (A) Cerebral cortex, (B) Hippocampus, (C) Thalamus, (D) Cerebellum and (E) Brainstem. Rats received saline (0.5 mL) or xylazine (5.2 mg/kg) intraperitoneally and then were sacrificed 10, 10, 20, 40 or 60 min later for control, Xyl1, Xyl2, Xyl3 or Xyl4, respectively. Western blot analyses were performed with anti-AMPKα and anti-phosphor-AMPKα (Thr172). Data for densitometry represent the mean ± SEM obtained from six independent series of Western blotting for each animal group and time point after the procedure. * P < 0.05, ** P < 0.01 vs control. Representative blots are shown below graph.

We also examined the phosphorylation of AMPKα at Thr172. The brain tissues from rats that were intraperitoneally injected with saline or xylazine were subjected to western blot analysis using the anti-phospho-AMPKα (Thr172) and anti AMPKα antibodies (Fig 5). In the cerebral cortex, phosphorylated AMPKα was slightly increased and peaked in the Xyl3 group at 40 min (P < 0.01) after drug administration, and then decreased. Levels remained elevated in the Xyl4 group (Fig 5A). Phosphorylation levels of AMPKα began to increase in the Xyl3 group and decline in the Xyl4 group in the hippocampus (Fig 5B). Thalamic levels of phosphorylated AMPKα increased at 40 min and then returned to control levels at 60 min (Fig 5C). In the cerebellum, phosphorylated AMPKα levels peaked at 20 min in Xyl2 rats and recovered in Xyl4 rats (Fig 5D). Levels of phosphorylated AMPKα did not significantly change in Xyl1 or Xyl2 rats compared with control. Phosphorylation levels decreased in the brainstem at 60 min in the Xyl4 group (P < 0.05, Fig 5E).

## Discussion

It was proposed that adrenoceptors and cholinoceptors, which have properties in common with classical α-adrenoceptors, were involved in the anti-nociceptive action of xylazine [25]. The anti-nociceptive effect of systemic, intrathecal or intracerebroventricular administration of xylazine has been reported [26]. It was shown that the spinal and systemic antinociceptive effects of xylazine are dependent on an α2-adrenoceptor-mediated, and not on an opioid-mediated mechanism [27]. At an individual level, conditioned pain modulation was related to blood oxygenation level-dependent responses in human secondary somatosensory cortices [28]. Moreover, the xylazine-induced reduction in cerebral blood flow could explain the reduced brain oxygenation observed in ketamine-xylazine anesthetized rats [29]. It has also been demonstrated that xylazine inhibits the release of cytokines and chemokines and thus contributes to the down-regulation of inflammatory pain and hyperalgesia [30]. However, the role analgesics play in regulating signal transduction has not been fully elucidated. The present work investigated this hypothesis at both the gene and protein level.

Gene expression and regulation is an important part in signal transduction. By using spatially and temporally regulated transcription factors in a concentration-dependent fashion, genes are able to be expressed in a precise, temporally and spatially controlled manner [31]. Therefore, we measured the mRNA levels of LKB1, AMPKα1, and AMPKα2 following treatment with xylazine in time and space. AMPK activity in neurons is regulated mainly via LKB1 in mice and rats [32]. LKB1 is a serine/threonine protein kinase that is highly expressed in the neurons of rats [33]. It is reported that mRNA levels of LKB1 and AMPKα decrease significantly in response to fasting, and that refeeding normalizes this effect [34]. The results of our study showed that, compared with the sedative state, xylazine induced a significant decrease in the mRNA levels of LKB1 in the brainstem at 40 min after rats received xylazine, whereas a significant increase was observed in the cerebral cortex, hippocampus, thalamus, and cerebellum, suggesting that a region-dependent regulation mechanism may modulate LKB1 gene expression at the transcriptional level in rats exposed to xylazine. However, it was showed that the rapid increase of AMPKα gene expression was necessary, but not sufficient to induce tissue plasminogen activator [35], which may affect synaptic plasticity in glucose-deprived rat primary astrocytes. Moreover, xylazine restrained the excitement of the nervous system via increasing LKB1-AMPK gene expression in the cerebral cortex, hippocampus, thalamus and cerebellum, but decreased LKB1-AMPK gene expression in the brainstem. Furthermore, bestatin improved the peripheral antinociceptive effect of xylazine, suggesting the mobilization of endogenous opioid peptides since the presence of mRNA or protein have been observed in the synthesis of opioid peptides [36]. Although the mechanisms by which LKB1 or AMPK regulate downstream protein translation in the rat CNS is not clear, the data from the present study suggests that the AMPK signaling pathway may be one of the key regulators involved in the xylazine-induced analgesic effect in the CNS of rats. These results indicate that xylazine may play an important role in influencing gene expression, and thus, more efforts should be made at the molecular level for future research.

Regulation of gene expression includes a wide range of mechanisms that are used by cells to increase or decrease the production of specific gene products such as proteins [37]. To further confirm the results of mRNA expression changes, we performed western blot analysis of different rat brain regions. LKB1 was identified as a major upstream kinase in the AMPK cascade [16]. Rat AMPK kinase activity was found to copurify with LKB1, and this activity was immunoprecipitated using anti-LKB1 antibodies [38]. A previous study reported that acute administration of bromocriptine inhibits glucose-stimulated insulin secretion by an AMPK-dependent mechanism involving direct activation of the α2-adrenergic receptor [39]. Moreover, it was reported that downregulation of CaMKK2 activity markedly reduced AMPK activation in HeLa cells and that the expression of CaMKK2 in chemokine (C-C motif) ligand 13 cells, which did not express LKB1, caused a dramatic increase in AMPK activity [38]. Other studies demonstrated that phosphorylation at Thr-172-AMPK increased with a concurrent increase in the phosphorylation of Ser-428-LKB1 in the rat [32, 40]. Taken together, these findings suggest that it is possible that AMPK activation is a consequence of an α2-adrenergic receptor-mediated effect and may involve LKB1. The present results provide evidence that the transcriptional activation of the LKB1-AMPK signaling pathway needs not only phosphorylation of LKB1 at Ser428, but also phosphorylation of AMPKα at Thr172 in the rat brain following treatment with xylazine. Furthermore, other upstream kinases need to be considered in the activation of AMPK in the brain in sedated rats. In this study, we examined the regulation of AMPK by phosphorylation in rats and found that phosphorylation at Thr172 of AMPK increased with a concurrent increase in the phosphorylation of Ser428 of LKB1 in the rat cerebral cortex, hippocampus, thalamus and cerebellum after an intraperitoneal xylazine injection, whereas the phosphorylation of Thr-172-AMPKα, as well as the phosphorylation of Ser-428-LKB1, began to decrease at 20 min in the Xyl2 rat brainstem. The similar trend in LKB1 activity and AMPK activity indicated that a LKB1-AMPK signaling pathway may exist in the brain structures of rats. However, the most marked increase in AMPK phosphorylation was observed at 40 min in the Xyl3 cerebral cortex, hippocampus and thalamus, while the phosphorylation of LKB1 reached a maximal level at 20 min in Xyl2 rats in the corresponding regions. These results, which indicate that changes in p-LKB1 are not always closely associated with parallel alterations in p-AMPK, suggest that LKB1 is not the sole upstream regulator of AMPK activation [34, 41]. These studies suggest that AMPK kinase activity in the rat corresponds to LKB1, but it does not rule out the possibility that other AMPK activities exist in different tissues.

## Conclusion

In summary, our study showed that xylazine administration altered mRNA expression and protein phosphorylation levels of AMPK signaling molecules, suggesting that the LKB1-AMPK pathway plays a role in the sedative and tranquilizing effects in the CNS caused by xylazine treatment. These results are the first to suggest that the central analgesic effect of xylazine is associated with the LKB1-AMPK signal transduction pathway. The results of this work contribute to a greater understanding of the central analgesia mechanisms of a drug widely used in veterinary anaesthesia and analgesia. However, more work needs to be done to elucidate the relationship between xylazine and the signal transduction pathways.

## Supporting Information

S1 Table Effect of xylazine administration on the mRNA levels of LKB1 in rats. Rats received saline (0.5 mL) or xylazine (5.2 mg/kg) intraperitoneally and then were sacrificed 10, 10, 20, 40 or 60 min later for control, Xyl1, Xyl2, Xyl3 or Xyl4, respectively. Total RNA was isolated and subjected to real-time PCR analysis. The relative expression levels of mRNA were analyzed using the 2^−ΔΔCt^ method. Each value of the expression levels of LKB1 was normalized to the expression levels of β-actin. The mean mRNA expression ratio in the control group was designated as one. Statistical analyses were performed using one-way ANOVA followed by Tukey's post hoc tests.
(DOC)

S2 Table Effect of xylazine administration on the mRNA levels of AMPKα1 in rats. Rats received saline (0.5 mL) or xylazine (5.2 mg/kg) intraperitoneally and then were sacrificed 10, 10, 20, 40 or 60 min later for control, Xyl1, Xyl2, Xyl3 or Xyl4, respectively. Total RNA was isolated and subjected to real-time PCR analysis. The relative expression levels of mRNA were analyzed using the 2^−ΔΔCt^ method. Each value of the expression levels of AMPKα1 was normalized to the expression levels of β-actin. The mean mRNA expression ratio in the control group was designated as one. Statistical analyses were performed using one-way ANOVA followed by Tukey's post hoc tests.
(DOC)

S3 Table Effect of xylazine administration on the mRNA levels of AMPKα2 in rats. Rats received saline (0.5 mL) or xylazine (5.2 mg/kg) intraperitoneally and then were sacrificed 10, 10, 20, 40 or 60 min later for control, Xyl1, Xyl2, Xyl3 or Xyl4, respectively. Total RNA was isolated and subjected to real-time PCR analysis. The relative expression levels of mRNA were analyzed using the 2^−ΔΔCt^ method. Each value of the expression levels of AMPKα2 was normalized to the expression levels of β-actin. The mean mRNA expression ratio in the control group was designated as one. Statistical analyses were performed using one-way ANOVA followed by Tukey's post hoc tests.
(DOC)

S4 Table Effect of xylazine administration on the levels of phosphorylated LKB1 in rats. Rats received saline (0.5 mL) or xylazine (5.2 mg/kg) intraperitoneally and then were sacrificed 10, 10, 20, 40 or 60 min later for control, Xyl1, Xyl2, Xyl3 or Xyl4, respectively. Western blot analyses were performed with anti-LKB1 and anti-phospho-LKB1 (Ser428). Data for densitometry were obtained from six independent series of Western blotting for each animal group and time point after the procedure. Densitometric analysis of p- LKB1 to LKB1 is represented as an arbitrary unit, normalized by β-actin. Statistical analyses were performed using one-way ANOVA followed by Tukey's post hoc tests.
(DOC)

S5 Table Effect of xylazine administration on the levels of phosphorylated AMPKα in rats. Rats received saline (0.5 mL) or xylazine (5.2 mg/kg) intraperitoneally and then were sacrificed 10, 10, 20, 40 or 60 min later for control, Xyl1, Xyl2, Xyl3 or Xyl4, respectively. Western blot analyses were performed with anti-AMPKα and anti-phosphor-AMPKα (Thr172). Data for densitometry were obtained from six independent series of Western blotting for each animal group and time point after the procedure. Densitometric analysis of p-AMPKα to AMPKα is represented as an arbitrary unit, normalized by β-actin. Statistical analyses were performed using one-way ANOVA followed by Tukey's post hoc tests.
(DOC)
