# Supplementary material for: Xylazine Activates Adenosine Monophosphate-Activated Protein Kinase Pathway in the Central Nervous System of Rats
Source: PLoS One. 2016 Apr 6;11(4):e0153169. doi: 10.1371/journal.pone.0153169 (PMC4822969; doi:10.1371/journal.pone.0153169)
Supplement: S1 Table — Rats received saline (0.5 mL) or xylazine (5.2 mg/kg) intraperitoneally and then were sacrificed 10, 10, 20, 40 or 60 min later for control, Xyl1, Xyl2, Xyl3 or Xyl4, respectively. Total RNA was isolated and subjected to real-time PCR analysis. The relative expression levels of mRNA were analyzed using the 2−ΔΔCt method. Each value of the expression levels of LKB1 was normalized to the expression levels of β-actin. The mean mRNA expression ratio in the control group was designated as one. Statistical analyses were performed using one-way ANOVA followed by Tukey's post hoc tests. (DOC) [file pone.0153169.s001.doc]

**S1 Table. Effect of xylazine administration on the mRNA levels of LKB1 in rats.** Rats received saline (0.5 mL) or xylazine (5.2 mg/kg) intraperitoneally and then were sacrificed 10, 10, 20, 40 or 60 min later for control, Xyl1, Xyl2, Xyl3 or Xyl4, respectively. Total RNA was isolated and subjected to real-time PCR analysis. The relative expression levels of mRNA were analyzed using the 2^−ΔΔCt^ method. Each value of the expression levels of LKB1 was normalized to the expression levels of β-actin. The mean mRNA expression ratio in the control group was designated as one. Statistical analyses were performed using one-way ANOVA followed by Tukey's post hoc tests.

| Brain regions | Control | Xyl1 | Xyl2 | Xyl3 | Xy4 |
| --- | --- | --- | --- | --- | --- |
| Cerebral cortex | 1 | 1.28 ± 0.12 | 2.22 ± 0.34^**^ | 4.94 ± 0.19^**^ | 3.72 ± 0.17^**^ |
| Hippocampus | 1 | 0.77 ± 0.10 | 1.08 ± 0.14 | 5.20 ± 0.40^**^ | 5.15 ± 0.31^**^ |
| Thalamus | 1 | 1.58 ± 0.19 | 1.35 ± 0.23 | 1.62 ± 0.25 | 4.14 ± 0.16^**^ |
| Cerebellum | 1 | 0.79 ± 0.09 | 2.63 ± 0.16^**^ | 3.33 ± 0.20^**^ | 4.17 ± 0.31^**^ |
| Brainstem | 1 | 0.84 ± 0.04^*^ | 0.34 ± 0.06^**^ | 0.27 ± 0.03^**^ | 0.19 ± 0.02^**^ |

LKB1, liver kinase B1. Data are expressed as means ± SEM (n = 6). *P < 0.05, **P < 0.01 compared with the control group.
