# Supplementary material for: Xylazine Activates Adenosine Monophosphate-Activated Protein Kinase Pathway in the Central Nervous System of Rats
Source: PLoS One. 2016 Apr 6;11(4):e0153169. doi: 10.1371/journal.pone.0153169 (PMC4822969; doi:10.1371/journal.pone.0153169)
Supplement: S2 Table — Rats received saline (0.5 mL) or xylazine (5.2 mg/kg) intraperitoneally and then were sacrificed 10, 10, 20, 40 or 60 min later for control, Xyl1, Xyl2, Xyl3 or Xyl4, respectively. Total RNA was isolated and subjected to real-time PCR analysis. The relative expression levels of mRNA were analyzed using the 2−ΔΔCt method. Each value of the expression levels of AMPKα1 was normalized to the expression levels of β-actin. The mean mRNA expression ratio in the control group was designated as one. Statistical analyses were performed using one-way ANOVA followed by Tukey's post hoc tests. (DOC) [file pone.0153169.s002.doc]

**S2 Table. Effect of xylazine administration on the mRNA levels of AMPKα1 in rats.** Rats received saline (0.5 mL) or xylazine (5.2 mg/kg) intraperitoneally and then were sacrificed 10, 10, 20, 40 or 60 min later for control, Xyl1, Xyl2, Xyl3 or Xyl4, respectively. Total RNA was isolated and subjected to real-time PCR analysis. The relative expression levels of mRNA were analyzed using the 2^−ΔΔCt^ method. Each value of the expression levels of AMPKα1 was normalized to the expression levels of β-actin. The mean mRNA expression ratio in the control group was designated as one. Statistical analyses were performed using one-way ANOVA followed by Tukey's post hoc tests.

| Brain regions | Control | Xyl1 | Xyl2 | Xyl3 | Xy4 |
| --- | --- | --- | --- | --- | --- |
| Cerebral cortex | 1 | 1.09 ± 0.09 | 1.52 ± 0.16 | 1.54 ± 0.17 | 2.66 ± 0.18^**^ |
| Hippocampus | 1 | 1.26 ± 0.18 | 2.59 ± 0.24^**^ | 4.10 ± 4.68^**^ | 4.68 ± 0.41^**^ |
| Thalamus | 1 | 0..92 ± 0.13 | 1.85 ± 0.17 | 2.43 ± 0.37^**^ | 3.34 ± 0.29^**^ |
| Cerebellum | 1 | 0.76 ± 0.10 | 0.98 ± 0.05 | 1.37 ± 0.20 | 2.56 ± 0.31^**^ |
| Brainstem | 1 | 1.07 ± 0.07 | 0.47 ± 0.04^**^ | 0.37 ± 0.02^**^ | 0.20 ± 0.02^**^ |

AMPKα1, adenosine 5’-monophosphate-activated protein kinase α1. Data are expressed as means ± SEM (n = 6). *P < 0.05, **P < 0.01 compared with the control group.
