# Supplementary material for: Xylazine Activates Adenosine Monophosphate-Activated Protein Kinase Pathway in the Central Nervous System of Rats
Source: PLoS One. 2016 Apr 6;11(4):e0153169. doi: 10.1371/journal.pone.0153169 (PMC4822969; doi:10.1371/journal.pone.0153169)
Supplement: S3 Table — Rats received saline (0.5 mL) or xylazine (5.2 mg/kg) intraperitoneally and then were sacrificed 10, 10, 20, 40 or 60 min later for control, Xyl1, Xyl2, Xyl3 or Xyl4, respectively. Total RNA was isolated and subjected to real-time PCR analysis. The relative expression levels of mRNA were analyzed using the 2−ΔΔCt method. Each value of the expression levels of AMPKα2 was normalized to the expression levels of β-actin. The mean mRNA expression ratio in the control group was designated as one. Statistical analyses were performed using one-way ANOVA followed by Tukey's post hoc tests. (DOC) [file pone.0153169.s003.doc]

**S3 Table. Effect of xylazine administration on the mRNA levels of AMPKα2 in rats.** Rats received saline (0.5 mL) or xylazine (5.2 mg/kg) intraperitoneally and then were sacrificed 10, 10, 20, 40 or 60 min later for control, Xyl1, Xyl2, Xyl3 or Xyl4, respectively. Total RNA was isolated and subjected to real-time PCR analysis. The relative expression levels of mRNA were analyzed using the 2^−ΔΔCt^ method. Each value of the expression levels of AMPKα2 was normalized to the expression levels of β-actin. The mean mRNA expression ratio in the control group was designated as one. Statistical analyses were performed using one-way ANOVA followed by Tukey's post hoc tests.

| Brain regions | Control | Xyl1 | Xyl2 | Xyl3 | Xy4 |
| --- | --- | --- | --- | --- | --- |
| Cerebral cortex | 1 | 1.25 ± 0.32 | 2.64 ± 0.22^**^ | 4.70 ± 0.53^**^ | 4.99 ± 0.60^**^ |
| Hippocampus | 1 | 1.20 ± 0.29 | 2.82 ± 0.17^**^ | 4.87 ± 0.51^**^ | 3.07 ± 0.12^**^ |
| Thalamus | 1 | 1.26 ± 0.10 | 1.86 ± 0.11^**^ | 1.32 ± 0.07 | 0.49 ± 0.08^**^ |
| Cerebellum | 1 | 0.75 ± 0.08 | 1.74 ± 0.18^**^ | 2.33 ± 0.13^**^ | 1.17 ± 0.12 |
| Brainstem | 1 | 1.05 ± 0.08 | 0.66 ± 0.05^**^ | 0.49 ± 0.04^**^ | 0.17 ± 0.02^**^ |

AMPKα2, adenosine 5’-monophosphate-activated protein kinase α2. Data are expressed as means ± SEM (n = 6). *P < 0.05, **P < 0.01 compared with the control group.
