# Supplementary material for: Xylazine Activates Adenosine Monophosphate-Activated Protein Kinase Pathway in the Central Nervous System of Rats
Source: PLoS One. 2016 Apr 6;11(4):e0153169. doi: 10.1371/journal.pone.0153169 (PMC4822969; doi:10.1371/journal.pone.0153169)
Supplement: S4 Table — Rats received saline (0.5 mL) or xylazine (5.2 mg/kg) intraperitoneally and then were sacrificed 10, 10, 20, 40 or 60 min later for control, Xyl1, Xyl2, Xyl3 or Xyl4, respectively. Western blot analyses were performed with anti-LKB1 and anti-phospho-LKB1 (Ser428). Data for densitometry were obtained from six independent series of Western blotting for each animal group and time point after the procedure. Densitometric analysis of p- LKB1 to LKB1 is represented as an arbitrary unit, normalized by β-actin. Statistical analyses were performed using one-way ANOVA followed by Tukey's post hoc tests. (DOC) [file pone.0153169.s004.doc]

**S4 Table. Effect of xylazine administration on the levels of phosphorylated LKB1 in rats.** Rats received saline (0.5 mL) or xylazine (5.2 mg/kg) intraperitoneally and then were sacrificed 10, 10, 20, 40 or 60 min later for control, Xyl1, Xyl2, Xyl3 or Xyl4, respectively. Western blot analyses were performed with anti-LKB1 and anti-phospho-LKB1 (Ser428). Data for densitometry were obtained from six independent series of Western blotting for each animal group and time point after the procedure. Densitometric analysis of p- LKB1 to LKB1 is represented as an arbitrary unit, normalized by β-actin. Statistical analyses were performed using one-way ANOVA followed by Tukey's post hoc tests.

| Brain regions | Control | Xyl1 | Xyl2 | Xyl3 | Xy4 |
| --- | --- | --- | --- | --- | --- |
| Cerebral cortex | 0.74 ± 0.04 | 0.97 ± 0.08 | 1.50 ± 0.13^**^ | 0.75 ± 0.10 | 0.83 ± 0.06 |
| Hippocampus | 0.44 ± 0.05 | 0.51 ± 0.07 | 1.53 ± 0.12^**^ | 0.70 ± 0.06 | 0.58 ± 0.09 |
| Thalamus | 0.38 ± 0.06 | 0.34 ± 0.09 | 2.26 ± 0.23^**^ | 0.28 ± 0.06 | 0.37 ± 0.08 |
| Cerebellum | 1.42 ± 0.27 | 4.67 ± 0.32^**^ | 7.58 ± 0.35^**^ | 4.78 ± 0.42^**^ | 1.20 ± 0.21 |
| Brainstem | 2.67 ± 0.11 | 2.80 ± 0.32 | 1.72 ± 0.23 | 1.40 ± 0.29^*^ | 1.21 ± 0.13^*^ |

LKB1, liver kinase B1. Data are expressed as means ± SEM (n = 6). *P < 0.05, **P < 0.01 compared with the control group.
