# Supplementary material for: Xylazine Activates Adenosine Monophosphate-Activated Protein Kinase Pathway in the Central Nervous System of Rats
Source: PLoS One. 2016 Apr 6;11(4):e0153169. doi: 10.1371/journal.pone.0153169 (PMC4822969; doi:10.1371/journal.pone.0153169)
Supplement: S5 Table — Rats received saline (0.5 mL) or xylazine (5.2 mg/kg) intraperitoneally and then were sacrificed 10, 10, 20, 40 or 60 min later for control, Xyl1, Xyl2, Xyl3 or Xyl4, respectively. Western blot analyses were performed with anti-AMPKα and anti-phosphor-AMPKα (Thr172). Data for densitometry were obtained from six independent series of Western blotting for each animal group and time point after the procedure. Densitometric analysis of p-AMPKα to AMPKα is represented as an arbitrary unit, normalized by β-actin. Statistical analyses were performed using one-way ANOVA followed by Tukey's post hoc tests. (DOC) [file pone.0153169.s005.doc]

**S5 Table. Effect of xylazine administration on the levels of phosphorylated AMPKα in rats.** Rats received saline (0.5 mL) or xylazine (5.2 mg/kg) intraperitoneally and then were sacrificed 10, 10, 20, 40 or 60 min later for control, Xyl1, Xyl2, Xyl3 or Xyl4, respectively. Western blot analyses were performed with anti-AMPKα and anti-phosphor-AMPKα (Thr172). Data for densitometry were obtained from six independent series of Western blotting for each animal group and time point after the procedure. Densitometric analysis of p-AMPKα to AMPKα is represented as an arbitrary unit, normalized by β-actin. Statistical analyses were performed using one-way ANOVA followed by Tukey's post hoc tests.

| Brain regions | Control | Xyl1 | Xyl2 | Xyl3 | Xy4 |
| --- | --- | --- | --- | --- | --- |
| Cerebral cortex | 0.32 ± 0.10 | 0.63 ± 0.11 | 0.74 ± 0.12 | 1.91 ± 0.22^**^ | 1.32 ± 0.28 |
| Hippocampus | 1.03 ± 0.22 | 0.54 ± 0.23 | 0.85 ± 0.22 | 3.95 ± 0.57^**^ | 2.98 ± 0.22^*^ |
| Thalamus | 0.44 ± 0.04 | 0.46 ± 0.07 | 1.19 ± 0.12^**^ | 1.49 ± 0.11^**^ | 0.32 ± 0.04 |
| Cerebellum | 0.34 ± 0.06 | 1.06 ± 0.13^*^ | 2.19 ± 0.16^**^ | 1.98 ± 0.19^**^ | 0.64 ± 0.18 |
| Brainstem | 4.54 ± 0.33 | 4.89 ± 0.48 | 4.43 ± 0.26 | 3.12 ± 0.19 | 1.46 ± 0.20^*^ |

AMPKα, adenosine 5’-monophosphate-activated protein kinase α. Data are expressed as means ± SEM (n = 6). *P < 0.05, **P < 0.01 compared with the control group.
